# IGFBP2 secretion by mammary adipocytes limits breast cancer invasion

**DOI:** 10.1126/sciadv.adg1840

**Published:** 2023-07-12

**Authors:** James R. W. Conway, Defne D. Dinç, Gautier Follain, Oona Paavolainen, Jasmin Kaivola, Pia Boström, Pauliina Hartiala, Emilia Peuhu, Johanna Ivaska

**Affiliations:** ^1^ Turku Bioscience Centre, University of Turku and Åbo Akademi University, FI-20520 Turku, Finland.; ^2^ Institute of Biomedicine, and Cancer Research Laboratory FICAN West, University of Turku, FI-20520 Turku, Finland.; ^3^ Western Finnish Cancer Center (FICAN West), University of Turku and Turku University Hospital, FI-20520 Turku, Finland.; ^4^ Department of Pathology, Turku University Hospital, 20520 Turku, Finland; University of Turku, 20520 Turku, Finland.; ^5^ Department of Plastic and General Surgery, Turku University Hospital, 20520 Turku, Finland.; ^6^ Medicity Research Laboratory, InFLAMES Research Flagship, University of Turku, Turku, Finland.; ^7^ Department of Life Technologies, University of Turku, FI-20520 Turku, Finland.; ^8^ InFLAMES Research Flagship Center, University of Turku, Turku, Finland.; ^9^ Foundation for the Finnish Cancer Institute, Tukholmankatu 8, FI-00014 Helsinki, Finland.

## Abstract

The progression of noninvasive ductal carcinoma in situ to invasive ductal carcinoma for patients with breast cancer results in a significantly poorer prognosis and is the precursor to metastatic disease. In this work, we have identified insulin-like growth factor–binding protein 2 (IGFBP2) as a potent adipocrine factor secreted by healthy breast adipocytes that acts as a barrier against invasive progression. In line with this role, adipocytes differentiated from patient-derived stromal cells were found to secrete IGFBP2, which significantly inhibited breast cancer invasion. This occurred through binding and sequestration of cancer-derived IGF-II. Moreover, depletion of IGF-II in invading cancer cells using small interfering RNAs or an IGF-II–neutralizing antibody ablated breast cancer invasion, highlighting the importance of IGF-II autocrine signaling for breast cancer invasive progression. Given the abundance of adipocytes in the healthy breast, this work exposes the important role they play in suppressing cancer progression and may help expound upon the link between increased mammary density and poorer prognosis.

## Teaser

Adipocyte-derived IGFBP2 from healthy stroma effectively limits IGF-II-driven breast cancer progression.

## INTRODUCTION

As breast cancer incidence continues to increase globally, the progression to metastatic disease remains the leading cause of death (*1*). Metastasis is a multistep process, beginning with a switch from a noninvasive ductal carcinoma to an invasive phenotype at the primary site. Extensive research into the trigger for this switch has revealed both cancer cell–intrinsic and –extrinsic drivers, where reciprocal signaling between the extracellular environment and the tumor leads to a concomitant progression of both stromal and cancer cells toward an aggressive disease state (*2*). Healthy mammary gland stroma has a sparse extracellular matrix (ECM) and is dominated by an abundance of adipocytes (*3*). Conversely, the breast cancer microenvironment is characterized by a high degree of desmoplasia and a reduced number and size of adipocytes adjacent to the tumor (*4*, *5*). It has been shown that changes in the composition and architecture of the mammary ECM during breast cancer development support invasive progression, and loss of this ECM can result in reversion to a less-aggressive disease state (*6*, *7*). While the importance of the ECM is clear, the established link between increased mammary density and a higher risk of breast cancer development has, so far, failed to address the role of adipocytes in containing the disease (*3*). Adipose tissue is a significant endocrine organ, and secretion of adipocrine factors plays a key role in tissue homeostasis (*4*). Notably, the processes through which tumors are able to overwhelm the homeostatic mechanisms aimed at their containment remains poorly explored. In this work, we uncover a mechanism by which mammary adipocytes provide a barrier to cancer invasive progression and, through antibody-based therapeutic intervention, suggest possible routes for reintroduction of this mechanism into a clinical setting.

## RESULTS

### HUVEC-derived angiocrine factor(s) reduce breast cancer invasion

**Fig. 1.**
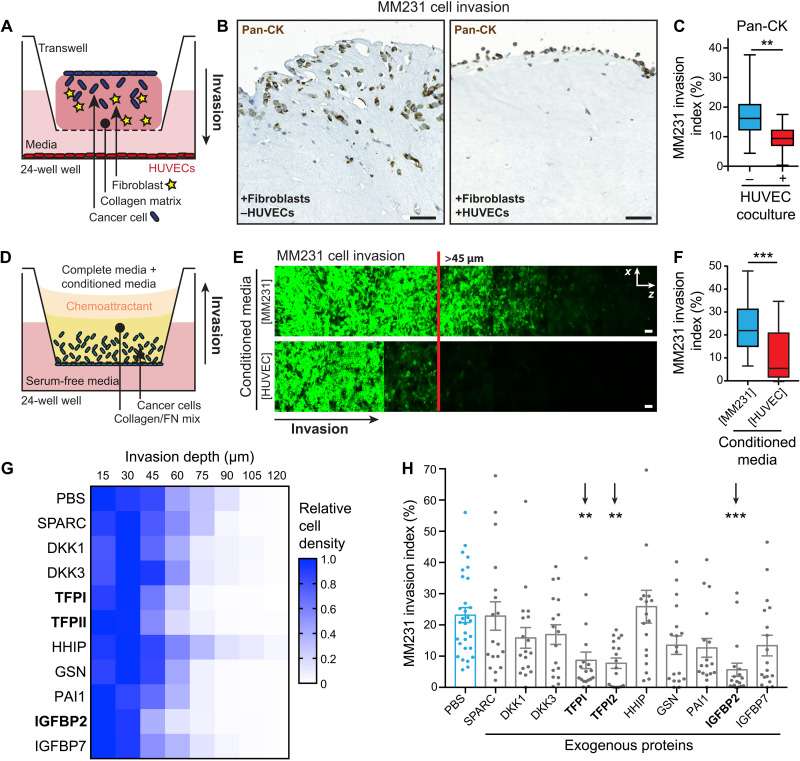
HUVECs secrete an anti-invasive factor effective against MM231 breast cancer cells. (**A**) Schematic of the fibroblast-contracted three-dimensional (3D) collagen matrix cancer cell invasion assay with HUVEC coculture. (**B** and **C**) Representative images (B) and quantification (C) of MM231 cell [stained with pan-cytokeratin (Pan-CK); epithelial cell marker] invasion into fibroblast-contracted 3D collagen matrices ± HUVEC coculture, performed in the presence of endothelial growth factor-reduced medium. Scale bars, 50 μm. [*n* = 3 biological replicates, triplicate matrices, eight regions per condition per replicate; one-way analysis of variance (ANOVA) with Tukey correction; **P < 0.01 and ***P < 0.001.] (**D**) Schematic of the inverted invasion assay platform. (**E** and **F**) Representative images (E) and quantification (F) of MM231 breast cancer cell (in green) invasion into inverted collagen/fibronectin matrices in the presence of concentrated conditioned media added to the normal culture medium (origin specified in square brackets). Scale bars, 50 μm. *n* = 3 biological replicates performed in triplicate, with three stacks per transwell; two-tailed Student’s *t* test with Welch’s correction; ***P < 0.001. (**G** and **H**) Inverted invasion screen heatmap (G) and quantification (H) of MM231 cell invasion in the presence of the indicated exogenously administered recombinant proteins (5 μM; *n* = 3 biological replicates performed with duplicate transwells per condition, with three stacks per transwell; one-way ANOVA with Tukey correction; **P < 0.01 and ***P < 0.001).

Cancer cell invasion away from the primary tumor commonly leads to intravasation into the adjacent blood and lymphatic vessels (*8*). To model this process in vitro, we assessed the invasion of cancer cells into fibroblast-contracted collagen I matrices toward human umbilical vein endothelial cells (HUVECs; Fig. 1A) (*9*). The presence of proximal endothelial cells did not promote the invasion of the MDA-MB-231 (MM231) triple-negative breast cancer cells (TNBCs), but instead markedly inhibited this process (Fig. 1B; quantified in Fig. 1C), while having no effect on proliferation (fig. S1, A and B). This indicated the presence of an anti-invasive angiocrine factor(s) secreted by the HUVECs. To confirm this hypothesis, we applied HUVEC- or MM231-conditioned medium to an inverted invasion assay platform (Fig. 1D) and found again that the presence of the HUVEC-derived angiocrine factor(s) markedly reduced the invasion of MM231 cancer cells (Fig. 1, E and F).

Endothelial cells from other tissues [conditioned medium from immortalized dermal microvascular endothelial, HMEC-1, cell line or primary human pulmonary microvascular endothelial cells (HPMECs)] did not secrete the same anti-invasive factor(s) as the HUVECs (fig. S1, C to F), suggesting that this could be a HUVEC-specific secreted factor(s), rather than a general feature of endothelia. In contrast to our work, others have shown that HUVECs cultured in three-dimensional (3D) microfluidic channels under flow induce the invasion of adjacent pancreatic cancer cells (*10*, *11*). However, the HUVECs cultured in our study are not exposed to flow-induced shear stress, which is known to change gene expression profiles and cell alignment (*12*), and are not in direct contact with breast cancer cells. These factors, along with the cancer type, are most likely the key differentiating features between our study and prior work.

To determine the secreted angiocrine factor(s) responsible for the observed HUVEC-mediated anti-invasive effect, we selected four published HUVEC secretomes and found >296 common factors between two or more secretomes (data S1) (*13*–*16*). These were then filtered through the tumor suppressor gene database (TSGene 2.0) (*17*) to identify candidates with putative antitumor activity. This analysis yielded a list of 10 candidate anti-invasive angiocrine factors with previous links to hedgehog [hedgehog-interacting protein (HHIP)], Wnt [Dickkopf-related protein 1 (DKK1) and DKK2], insulin-like growth factor (IGF) regulation [IGF-binding protein 2 (IGFBP2) and IGFBP7], or calcium [secreted protein acidic and cysteine rich (SPARC)] signaling, as well as protease inhibition [plasminogen activator inhibitor 1 (PAI1)], actin polymerization [gelsolin (GSN)], and blood coagulation [tissue factor pathway inhibitor (TFPI and TFPI2)]. We then screened for potential effects on proliferation and invasion. While none of these factors significantly affected MM231 cell proliferation (fig. S1, G and H), the exogenous addition of TFPI, TFPI2, and IGFBP2 did significantly reduce MM231 cell invasion (Fig. 1, G and H).

### IGFBP2 secretion by HUVECs and fibroblasts exhibits a potent anti-invasive effect

**Fig. 2.**
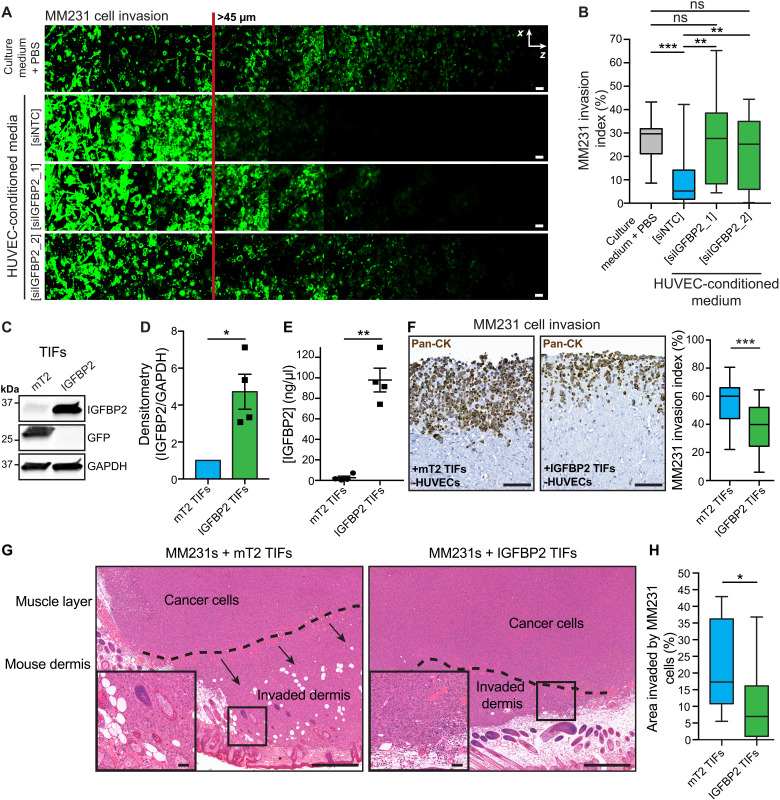
Stromal-derived IGFBP2 is sufficient to inhibit MM231 breast cancer cell invasion. (**A** and **B**) Representative images (A) and quantification (B) of MM231 breast cancer cell invasion in inverted collagen/fibronectin matrices in the presence of conditioned medium from HUVECs transfected with siRNAs against IGFBP2 (siIGFBP2_1 and siIGFBP2_2) or siNTC. Scale bars, 50 μm. *n* = 3 biological replicates performed in triplicate, with three stacksper transwell; one-way ANOVA with Tukey correction; **P < 0.01 and ***P < 0.001; ns, not significant. (**C** and **D**) Representative Western blot (C) and densitometry analysis (D) of TIFs stably overexpressing mTurquoise2 (mT2) or IGFBP2 (*n* = 4; one-sample *t* test; *P < 0.05). (**E**) Enzyme-linked immunosorbent assay (ELISA) for human IGFBP2 in concentrated conditioned media from TIFs overexpressing mT2 or IGFBP2, measured in duplicate (*n* = 3 biological replicates; two-tailed Student’s *t* test; **P < 0.01). (**F**) Representative images and quantification of fibroblast-contracted 3D collagen invasion assays of MM231 cancer cell invasion in mT2 or IGFBP2 TIF-contracted matrices after 14 days and stained for Pan-CK (brown). Scale bars, 100 μm. *n* = 3 biological replicates, triplicate matrices, eight regions per condition per replicate; one-way ANOVA with Tukey correction; ***P < 0.001. (**G** and **H**) Representative hematoxylin and eosin (H&E) images (G) and quantification (H) of MM231 cell invasion into the mouse dermis from subcutaneous xenografts of MM231 co-injected with TIFs overexpressing either mT2 (control) or IGFBP2. Scale bars, 500 μm; insets, 50 μm. *n* = 10 mice (mT2) or 12 mice (IGFBP2).

The tissue factor pathway has a well-established link with cancer progression, and particularly with metastatic dissemination (*18*), while the IGFBPs are reported to have conflicting roles in breast cancer progression (*19*–*22*). Linked to the observed HUVEC-specific anti-invasive effect, we next assessed the levels of IGFBP2 secreted by different endothelial cell lines. We found that it was significantly expressed in the HUVECs alone (fig. S2A), suggesting that the presence of IGFBP2 in the HUVEC-conditioned medium may be essential for the observed anti-invasive effect (fig. S1, C to F). To confirm this hypothesis, we assessed the anti-invasive effect of conditioned medium collected from *IGFBP2*-silenced HUVECs. After silencing, the secreted IGFBP2 was significantly reduced, compared to the nontarget small interfering RNA (siRNA) control (siNTC; fig. S2, B and C), and where IGFBP2 was no longer secreted, the anti-invasive effect was also lost (Fig. 2A; quantified in Fig. 2B).

In the 3D coculture assay, cancer cells invade into collagen that has been remodeled by fibroblasts. To further validate the anti-invasive effect of IGFBP2 and to model the putative outcome of secreted IGFBP2, we generated telomerase immortalized fibroblasts (TIFs) with stable overexpression and secretion of IGFBP2 or a control fluorescent protein construct [mTurquoise2 (mT2); Fig. 2, C to E]. Consistent with earlier findings, the MM231 cells were significantly less efficient at invading into 3D collagen I matrices contracted by IGFBP2-secreting TIFs, compared to control mT2 TIFs (Fig. 2F), demonstrating that secreted IGFBP2 is anti-invasive. Concordant with the earlier results, TIF-secreted IGFBP2 had no detectable effect on the proliferation of MM231 cells (fig. S2D).

To assess the effect of IGFBP2 reintroduction on the breast tumor microenvironment, we co-xenografted MM231s with the IGFBP2- or mT2-overexpressing TIFs (Fig. 2G). In this mouse model, we observed no effect on the proliferation of cancer cells (fig. S2E). However, there was a clear reduction in cancer cell invasion into the surrounding stroma in the IGFBP2 TIF co-xenografts (Fig. 2H), supporting the idea that IGFBP2 plays a protective role in the stroma and, when present, is able to contain the tumor.

### Mammary stromal adipocytes secrete IGFBP2 and reduce breast cancer invasion

**Fig. 3.**
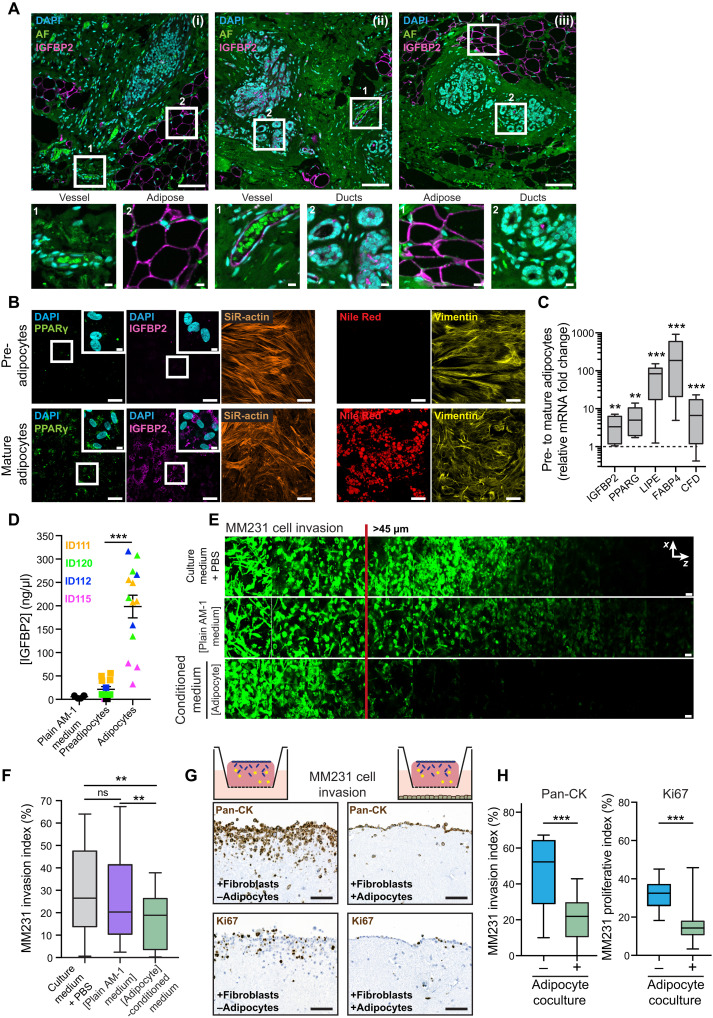
Breast adipocytes secrete the anti-invasive factor IGFBP2. (**A**) Three representative images [(i) to (iii)] of human breast tissue stained for IGFBP2 (magenta) and counterstained with 4′,6-diamidino-2-phenylindole (DAPI) (cyan). Autofluorescence (AF) signal is green. Scale bars, 100 μm; insets, 10 μm. *n* = 2 adjacent healthy breast tissue sections from ductal carcinoma in situ (DCIS) patient samples. (**B**) Representative images of primary human breast pre- and mature adipocytes with immunofluorescence staining for IGFBP2/PPARγ/actin or Nile Red/vimentin. Scale bars, 50 μm; insets, 10 μm. *n* = 4 normal reduction mammoplasty patient samples from which preadipocytes were isolated, cultured, and differentiated into mature adipocytes. (**C**) Fold change in gene expression between pre- and mature adipocytes, normalized to glyceraldehyde-3-phosphate dehydrogenase (*GAPDH*), and detected by quantitative reverse transcription polymerase chan reaction [adipocytes from *n* = 4 patient samples, differentiated as in (B), in triplicate; one-sample *t* test; **P < 0.01 and ***P < 0.001]. *PPARG*, peroxisome proliferator activated receptor gamma; *LIPE*, lipase E; *FABP4*, fatty acid binding protein 4; *CFD*, complement factor D. (**D**) ELISA for human IGFBP2 from pre- and mature adipocyte conditioned media, compared to the adipocyte culture media [AM-1; adipocytes from *n* = 4 patient samples processed as in (B) and media collected; two-tailed Student’s *t* test with Welch’s correction; ***P < 0.001]. (**E** and **F**) Representative images (E) and quantification (F) of MM231 cell invasion in inverted collagen/fibronectin matrices in the presence of concentrated conditioned media from adipocytes or adipocyte growth medium, AM-1, or full culture medium with an equivalent volume of PBS added. Scale bars, 50 μm. adipocytes from *n* = 4 patient samples, processed as in (B) and media collected, performed in triplicate with three stacks per transwell; one-way ANOVA with Tukey correction; **P < 0.01. (**G** and **H**) Representative images (G) and quantification (H) of a fibroblast-contracted 3D collagen matrix cancer cell invasion assay monitoring MM231 cancer cell invasion ± mature adipocyte coculture and stained for either Pan-CK or the proliferation marker Ki67. Scale bars, 100 μm. *n* = 3 biological replicates, triplicate matrices, eight regions per condition per replicate; one-way ANOVA with Tukey correction; ***P < 0.001).

Changes in the breast stroma are an essential factor in breast cancer progression. This prompted us to stain patient samples for IGFBP2 in adjacent healthy breast tissue sections to judge the physiological relevance of our findings to invasive progression (Fig. 3A; patient details in data S2). Unexpectedly, this staining revealed a strong positive IGFBP2 signal in the adipocyte cells, whereas the signal exhibited by the vessels was lower or negative (Fig. 3A, i and ii). Similarly, when comparing the epithelial cells in the mammary ducts, it was again clear that the adipocytes were the main cells in the breast microenvironment that expressed IGFBP2 (Fig. 3A, iii). Together, these data suggest that adipocytes in the mammary stroma are the most prominent expressors of IGFBP2 in situ.

Having identified adipocytes as the cells in the mammary stroma with the highest expression of IGFBP2, we next investigated whether IGFBP2 was an adipocrine factor secreted into the breast microenvironment. Several groups have isolated primary adipocytes from patients (*23*, *24*). However, because of the scarcity of samples and the challenges associated with applying these protocols, we instead sought to differentiate isolated breast preadipocytes into mature adipocytes from human stromal samples collected from healthy reduction mammoplasty patients (see Materials and Methods for details). These differentiated primary patient cells were positive for the adipocyte marker peroxisome proliferator-activated receptor gamma (PPARγ) and the presence of lipid droplets, which marked the mature and not the preadipocyte cells (Fig. 3B). Similarly, the mature adipocytes showed an increased mRNA level of established adipocyte markers, when compared to preadipocytes (Fig. 3C). The mature adipocytes had a higher level of IGFBP2, which was visible in the immunofluorescence staining and at the mRNA level. This was consistent with an increase in secretion of IGFBP2 in conditioned medium from mature adipocytes (Fig. 3D). The secreted IGFBP2 had no effect on the proliferation of MM231 cells (fig. S2, F and G), but resulted in a significant reduction in cancer invasion, when compared to the plain medium [adipocyte growth medium (AM-1) or complete medium] controls (Fig. 3, E and F).

Lastly, coculture of cancer cells with the IGFBP2-secreting adipocytes significantly reduced cancer cell invasion into fibroblast-contracted 3D collagen I matrices (Fig. 3G; quantified in Fig. 3H). The addition of adipocytes into the coculture system also significantly reduced cancer cell proliferation (Fig. 3, G and H), suggesting that adipocrine factors other than IGFBP2 may be able to exert additional anticancer effects. Cumulatively, these findings uncover a role for healthy mammary stromal adipocytes in the containment of cancer invasion.

### IGFBP2 did not detectably bind to the cancer cell surface or ECM

We hypothesized that IGFBP2 could exert its anti-invasive function by binding to and inhibiting cell surface receptors. To test this, we tagged IGFBP2 with the clover fluorescent protein to enable visualization in the extracellular space. TIFs were then engineered to overexpress clover-tagged IGFBP2, which resulted in efficient IGFBP2 secretion and, when applied to invading MM231s (fig. S3, A and B), recapitulated the anti-invasive effects observed with the addition of untagged IGFBP2 (fig. S3, C and D). Having established the biological activity of the clover-tagged IGFBP2, we next applied the concentrated IGFBP2 medium (from the TIFs) to MM231 cells and assessed IGFBP2 binding to cancer cells using flow cytometry. With this approach, we observed no increase in the fluorescence signal in MM231 cells treated with clover-tagged or untagged (negative control) IGFBP2, compared to MM231s stably expressing IGFBP2-Clover (positive control; fig. S3, E and F). These experiments demonstrate that exogenous IGFBP2 does not bind to the surface of cancer cells, suggesting that the anti-invasive effects are occurring through binding of IGFBP2 to something in the cancer microenvironment.

Next, we explored the possibility of IGFBP2 binding to the ECM and interfering with cell-ECM interactions. It has previously been shown that IGFBP2 binds to the ECM through two domains, one in the linker domain and one in the C domain, which mediate binding to fibronectin, vitronectin, collagen IV, laminin, and heparin (*25*, *26*). To assess IGFBP2 binding to the ECM, we generated cell-derived matrices (CDMs) using TIFs stably expressing IGFBP2-Clover or mT2 (*27*, *28*). However, IGFBP2-Clover did not appear to decorate the collagen or laminin fibers, instead giving a weak nonspecific signal lower than the background signal observed with the mT2 control (fig. S3G). With this system, we also assessed the effect of IGFBP2 expression on ECM organization and fibronectin deposition within the collagen network and found no significant differences between the ECM generated by either mT2 or IGFBP2-Clover expressing fibroblasts (fig. S3, H to J). As CDMs involve a decellularization step that may have displaced the IGFBP2 from the CDM matrices, we also assessed the adhesion and spreading of MM231s on collagen/fibronectin in the presence of IGFBP2 and again found no significant effect (fig. S3, K and L). This led us to the conclusion that soluble rather than ECM-incorporated IGFBP2 is responsible for blocking invasion.

### IGFBP2 disrupts proinvasive IGF-II autocrine signaling

**Fig. 4.**
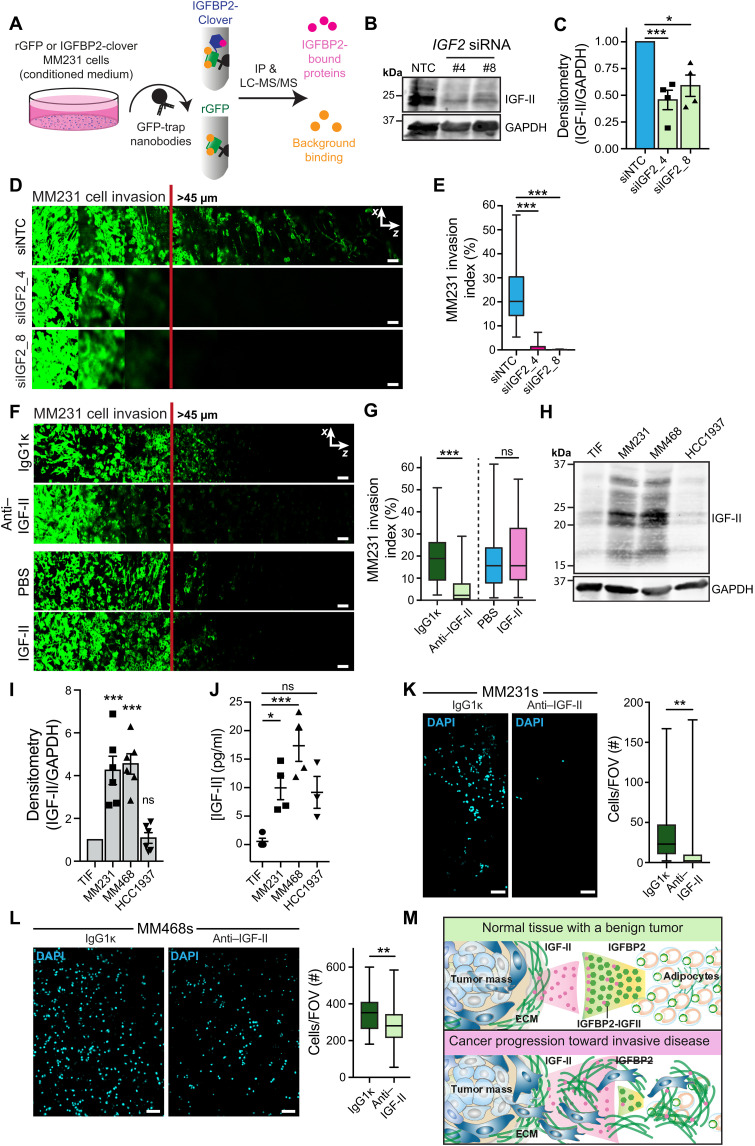
IGFBP2 acts through depletion of IGF-II from the cancer microenvironment. (**A**) Schematic of the proteomics experimental setup. LC-MS/MS, liquid chromatography–tandem mass spectrometry. IP, immunoprecipitation. (**B** and **C**) Representative Western blot (B) and densitometry analysis (C) after silencing of *IGF2* (IGF-II gene) using siRNAs in MM231 cells (*n* = 4 biological replicates; one-sample *t* test; *P < 0.05 and ***P < 0.001). (**D** and **E**) Representative images (D) and quantification (E) of MM231 cell invasion in inverted collagen/fibronectin matrices after *IGF2* silencing. Scale bars, 50 μm. *n* = 3 biological replicates, performed in duplicate with three stacks per transwell; one-way ANOVA with Tukey correction; ***P < 0.001. (**F** and **G**) Representative images (F) and quantification (G) of MM231 cell invasion in inverted collagen/fibronectin matrices treated with PBS, IGF-II (10 ng/ml), IgG1κ (10 μg/ml), or anti–IGF-II (10 μg/ml). Scale bars, 50 μm. *n* = 3 biological replicates, performed in duplicate with three stacks per transwell; one-way ANOVA with Tukey correction; ***P < 0.001. (**H** and **I**) Representative IGF-II Western blot (H) and quantification (I) of TIF, MM231, MM468, and HCC1937 cells (*n* = 6 biological replicates; one-sample *t* test; ***P < 0.001). (**J**) ELISA for human IGF-II in conditioned media from TIF, MM231, MM468, and HCC1937 cells (*n* = 4 biological replicates; one-way ANOVA with Dunnett’s correction; *P < 0.05 and ***P < 0.001). (**K** and **L**) Matrigel invasion assays for MM231 (K) and MM468 (L) cells treated with IgG1κ or anti–IGF-II (*n* = 3, eight fields of view (FOVs) per chamber, two to three invasion chambers per condition per replicate; two-tailed Student’s *t* test with Welch’s correction; **P < 0.01). Scale bars, 100 μm. (**M**) Schematic of the proposed mechanism for IGFBP2 inhibition of invasion through disruption of breast cancer IGF-II autocrine signaling.

Given the above results, we hypothesized that soluble IGFBP2 was binding to and sequestering a proinvasive molecule secreted by cancer cells. To explore this possibility, we performed unbiased mass spectrometry, incubating MM231 cells with conditioned medium from TIFs overexpressing IGFBP2-Clover or TIF-conditioned medium containing recombinant green fluorescent protein (rGFP; negative control). We then performed GFP-trap-immunoprecipitation and proteomics analysis of the medium to identify IGFBP2-binding partners (Fig. 4A and data S3). Only IGF-II was notably enriched in the IGFBP2 condition, and this corresponds to one of the two canonical binding partners of IGFBP2 (*25*, *26*).

To assess the role of IGF-II in MM231 invasion, we applied siRNAs to silence *IGF2* (Fig. 4B; quantified in Fig. 4C), and observed a clear reduction in invasion (Fig. 4D; quantified in Fig. 4E). Congruent with these data, sequestration of secreted IGF-II by exogenous administration of an anti–IGF-II antibody recapitulated the anti-invasive effect of IGFBP2 treatment (Fig. 4F; quantified in Fig. 4G). Treatment with recombinant IGF-II had no effect on invasion, suggesting that MM231s have already reached a proinvasive autocrine threshold (Fig. 4, F and G).

The anti–IGF-II antibody applied here has already shown promising efficacy as an anticancer agent in breast cancer xenografts (*29*). Given that TNBC remains the most aggressive breast cancer subtype with the poorest prognosis (*30*), we chose two additional TNBC cell lines, MDA-MB-468 (MM468) and HCC1937, to confirm the broader significance of IGF-II–driven invasion. We compared IGF-II levels across cell lines and found higher expression in MM468 and MM231 cells, compared to TIF and HCC1937 cells (Fig. 4H; quantified in Fig. 4I), which was paralleled by increased secretion (Fig. 4J). Notably, the pre- and mature adipocytes applied here also secrete IGF-II (fig. S4A), but at levels ~200-fold less than IGFBP2 (Fig. 3D), suggesting that IGFBPs are secreted in excess and likely completely sequester adipocyte-derived IGF-II in the extracellular space.

Breaching of the basement membrane is one of the first steps in breast cancer invasive progression, and when modeling this process using Matrigel invasion chambers, we again found a clear reduction in MM231 cell invasion in the presence of the anti–IGF-II function-blocking antibody and IGFBP2 (Fig. 4K). These results were recapitulated in the MM468s (Fig. 4L), which also showed high IGF-II expression (Fig. 4, H and I). In line with the IGFBP2 perturbations above, there was again no effect on proliferation when IGF-II was depleted from the extracellular space or when added exogenously (fig. S4, B to E). Similarly, when we applied MCF10DCIS.com (DCIS.com; fig. S4, F and G) breast cancer cells, which also express IGF-II, to the Matrigel invasion chambers (*31*), we again saw a significant reduction in invasion after IGFBP2 or anti–IGF-II treatments (fig. S4, H and I). Notably, DCIS.com cells undergo a ductal carcinoma in situ (DCIS) to invasive ductal carcinoma (IDC) transition when xenografted (*32*), suggesting that targeting IGF-II autocrine signaling may act to contain a wide range of breast cancers in situ. Cumulatively, we see that IGF-II depletion phenocopies IGFBP2 anti-invasive action in the TNBC and DCIS.com cell lines, supporting a model of stromal IGFBP2 disrupting proinvasive IGF-II autocrine signaling in cancer cells (Fig. 4M).

### IGFBP2-positive adipocytes are reduced in patient samples from DCIS and IDC

**Fig. 5.**
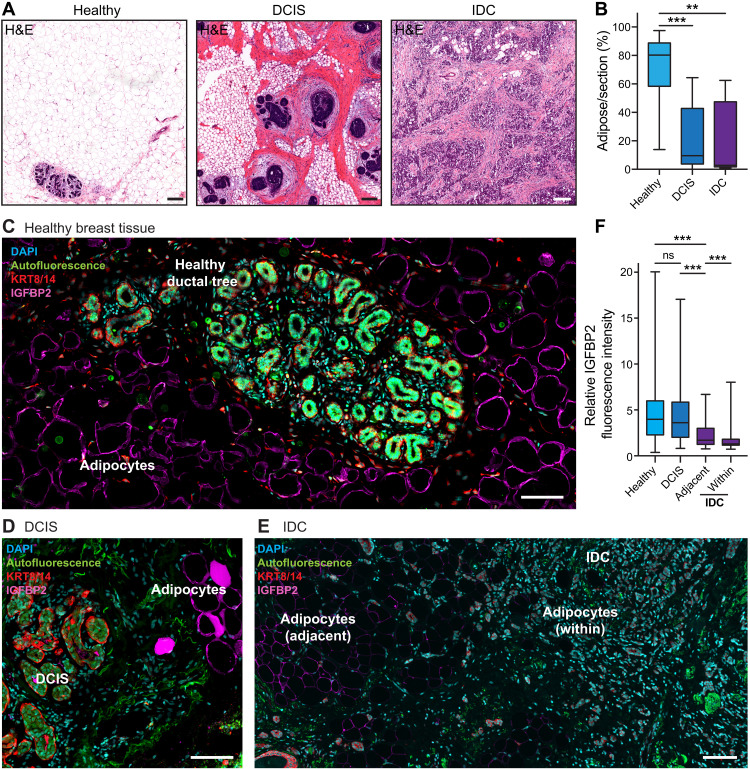
IGFBP2 expression during breast cancer progression. (**A**) Representative H&E-stained breast tissue samples from healthy patients, patients with DCIS, or patients with IDC. Scale bars, 200 μm. (**B**) Quantification of adipocytes per section from healthy (*n* = 8 patients, three to eight sections per patient), DCIS (*n* = 6 patients, one to four sections per patient), and IDC (*n* = 3 patients, one to two sections per patient). Kruskal-Wallis test using Dunn’s test to correct for multiple comparisons (**P < 0.01 and ***P < 0.001). (**C**) Representative image from a patient sample stained for IGFBP2 (magenta) and counterstained with DAPI (cyan) and keratin-8/keratin-14 (KRT8/14; red). Scale bar, 100 μm. *n* = 8 normal reduction mammoplasty patient samples. Autofluorescence is given in green. (**D**) Representative image of human DCIS breast cancer tissue stained for IGFBP2 (magenta) and counterstained with DAPI (cyan) and KRT8/14 (red). Scale bar, 100 μm (*n* = 4 DCIS patient samples). Autofluorescence is given in green. (**E**) Representative image of human IDC breast cancer tissue stained for IGFBP2 (magenta) and counterstained with DAPI (cyan) and KRT8/14 (red; *n* = 3 IDC patient samples). Scale bar, 100 μm. Autofluorescence is given in green. The brightness of IGFBP2 staining was increased for display purposes only. (**F**) Quantification of IGFBP2 per adipocyte from healthy (*n* = 8 patients, 143 to 280 adipocytes per patient), DCIS (*n* = 6 patients, 39 to 410 adipocytes per patient), and IDC (*n* = 3 patients, 75 to 283 adipocytes per patient). Kruskal-Wallis test using Dunn’s test to correct for multiple comparisons (***P < 0.001).

To further explore the clinical significance of these findings, we stained a cohort of healthy mammary gland, DCIS and IDC patient samples. High mammographic density is the strongest risk factor for breast cancer (*33*–*35*), where an increase in fibroglandular tissue occurs in concert with increased tissue stiffness, immune infiltration, and fibrosis (*36*, *37*). Increased density is paralleled by a reduction in adipose tissue, which also occurs during breast cancer progression (Fig. 5A; quantified in Fig. 5B). When we stained these patient samples, the number of IGFBP2-positive cells was severely reduced from healthy to disease states (Fig. 5, C to E). As the risk of breast cancer increases with age, we also investigated IGFBP2 expression in adipocytes in healthy breast tissue of younger (ages 18 to 26) and older (ages 40 to 45) patient cohorts (fig. S5). IGFBP2 expression, but not overall adiposity, was significantly lower in the older healthy patient cohort (fig. S5). Moreover, adipocytes in the IDC samples had significantly reduced IGFBP2 expression, compared to the DCIS and healthy samples (Fig. 5F), and this was further reduced in adipocytes contained within the tumor borders, compared to those at the invasive front (Fig. 5F). Together, these data are supportive of lower stromal IGFBP2 expression correlating with age-related cancer risk and disease progression. This IGFBP2 loss in the breast tissue microenvironment upon disease progression highlights that investigation of healthy stromal cells may provide key insights into the mechanisms of tissue homeostasis that is disrupted in cancer.

## DISCUSSION

Here we show that healthy mammary adipocytes are able to restrain breast cancer invasion through secretion of IGFBP2, a novel anti-invasive adipocrine factor. Our experiments demonstrate that a healthy human mammary gland is rich in IGFBP2-expressing adipocytes and that IGFBP2 secreted by normal tissue-derived adipocytes efficiently blocks breast cancer invasion. Our study describes an anti-invasive mechanism in which autocrine proinvasive IGF-II signaling by cancer cells is disrupted by IGFBP2 acting as a stromal sequester of IGF-II. IGFBP2 action could be efficiently mimicked with an IGF-II–neutralizing antibody, implying direct clinical and therapeutic implications of our discovery.

The tissue composition of human breast is unique, with glandular structures surrounded by abundant adipocytes and low tissue rigidity. In contrast, desmoplasia (increased deposition of ECM) and subsequent stiffening of the tumor microenvironment plays a key role in breast cancer aggression (*38*). Our discovery of the cancer-limiting activity of mammary gland adipocytes is original and focuses on an understudied aspect of breast cancer stroma. Previous studies into the role of adipocytes in breast cancer progression have primarily focused on their loss during the process (*3*) and the concept of increased tissue rigidity contributing to breast cancer invasion through mechanochemical signaling in cancer cells (*36*). In addition, recent work has explored the changes in mammary adipose tissue during tumor progression, finding that cancer-associated adipocytes express reduced adipogenesis-related genes, show increased browning, and secrete more inflammatory cytokines and matrix metalloproteinases, while furthering the desmoplastic reaction associated with breast cancer progression (*4*, *5*, *39*–*42*). In line with this work, we saw a loss of IGFBP2-positive adipocytes in DCIS and IDC patient samples, when compared to healthy breast tissue. Previous studies using cocultured adipocytes differentiated from 3T3 cells or adipose-derived stem cells have demonstrated growth-promoting and promigratory effects on MM231 and MCF7 cells in vitro (*43*–*45*). In contrast, we find that coculture of breast cancer cells with adipocytes differentiated from patient-derived stromal cells significantly inhibits invasion. These somewhat opposing results could be owing to the different source/state of the cells. Notably, recent work by Silva and colleagues (*46*) has demonstrated that lipofilling with healthy human adipose into mouse MCF7 xenografts reduces tumor growth and proliferation. Moreover, recent studies exploring the heterogeneity of stromal cells have motivated assessment of adipose tissue in a similar fashion (*47*) and have revealed far greater diversity than the general classifications of brown, white, and brite/beige (*48*–*50*). These findings highlight the significant impact that could be attained through restoration of IGFBP2 into the breast cancer microenvironment, along with an alternative perspective on the role of adipocytes in breast cancer progression.

IGFBP2 was identified as part of a screen for anti-invasive factors, where exogenous recombinant proteins were applied to invading MM231 breast cancer cells. IGFBP2 is involved in metabolic diseases (*19*, *51*). High-serum IGFBP2 is associated with a decreased risk of diabetes, playing a protective role against insulin resistance and obesity (*52*, *53*). Diabetes and obesity are associated with increased risk of breast cancer progression, as well as dysfunctional adipose tissue (*54*, *55*). Here, we found that IGFBP2 was able to bind and sequester IGF-II, which prevents the autocrine signaling of breast cancer cells and limits their progression toward invasive disease. However, the role of IGFBP2 in dysfunctional mammary adipocytes and the link to cancer is unclear, as it has been associated with both tumor progression and suppression (*19*, *56*). However, many studies consider only the intracellular IGFBP2 pool in cancer cells, where it appears to play a protumorigenic role (*19*–*21*). In contrast, exogenously applied IGFBP2 has been reported to inhibit in vitro and in vivo tumor growth (*57*), which is in line with IGF-II inhibition suppressing the growth of breast cancer xenografts (*29*). We failed to observe any IGFBP2- or anti–IGF-II–mediated effects on cell proliferation and detected only robust anti-invasive properties in our experimental models. Nevertheless, these studies and our work indicate an anticancer role for secreted extracellular IGFBP2 and underline the importance of differentiating between intracellular and extracellular pools when investigating IGFBPs.

While disruption of healthy adipose function supports breast cancer progression and local invasion into the co-opted tissue (*39*, *58*), approaches to recuperate or restore this adipose tissue could help restrain breast cancer progression before invasive disease. It has already been shown that the increased plasticity of breast cancer cells undergoing an epithelial-to-mesenchymal transition can be exploited to transdifferentiate them into adipocytes (*59*). This presents an exciting opportunity for the restoration of IGFBP2 into the cancer microenvironment to further contain the primary tumor. Furthermore, as high levels of *IGF2* have been linked to a poorer prognosis in breast cancer (*29*) and we find that IGF-II drives proinvasive autocrine signaling in breast cancer cells, this opens up a new avenue for therapeutic intervention through IGF-II depletion. In this way, this work places a spotlight on healthy stromal tissue in tumor containment and supports further investigation into IGFBPs in this process in other cancer subtypes.

## MATERIALS AND METHODS

### Animal experiments

Subcutaneous xenografts were performed with athymic nude mice (Foxn1^nu^; Envigo, UK) by co-injecting 6 × 10^6^ TIFs mT2/IGFBP2 and 2 × 10^6^ MM231 in phosphate-buffered saline (PBS). Tumors were then tracked by palpation with calipers until the tumor volume ($\frac{\mathrm{Length}\times {\mathrm{Width}}^{2}}{2}$) was >300 mm^3^, at which point the mice were euthanized and tumors were collected. Mice were housed in standard conditions (12-hour light/12-hour dark cycle) with food and water available ad libitum. All animal experiments were performed in accordance with the Finnish Act on Animal Experimentation (animal license number ESAVI/12558/2021). Quantification of adipose tissue in sections was performed in QuPath (*60*). Quantification of local invasion and Ki67-positive nuclei were also performed in QuPath on tumors that showed local invasion, as described previously (*61*), building upon the approach by quantifying the percentage of local invasion in the xenograft by selecting areas of invaded cells and dividing those by the total tumor area.

### CDM generation

CDMs were generated as described previously for TIFs (*28*), with only the modification of the TIFs themselves to stably express mT2 or IGFBP2. To assess changes in the fibronectin or collagen I fiber orientation in the presence of IGFBP2, CDMs were treated with recombinant CNA35-mCherry before fixation with 4% paraformaldehyde (PFA). These were then blocked overnight with 2.5% bovine serum albumin (BSA) (Sigma-Aldrich, A8022)/1 M glycine (ITW Reagents, A1067)/PBS before overnight staining with anti-fibronectin primary antibody (HFN7.1, mouse; Abcam, ab80923). Stained CDMs were then imaged on a spinning disk confocal microscope (3i Marianas CSU-W1; 40×/1.1 objective) and analyzed for staining density in FIJI [National Institutes of Health (NIH)] and fiber coherency using the OrientationJ package (FIJI).

### Cell line models

TIFs [a gift from J. C. Norman (Beatson Institute, Glasgow, Scotland, UK)], MM231 [American Type Culture Collection (ATCC), HTB-26], and MM468 (ATCC, HTB-132) cells were all cultured in Dulbecco’s modified Eagle’s medium (DMEM; Sigma-Aldrich, D2429) supplemented with 10% fetal bovine serum (FBS) and l-glutamine (100 mM). DCIS.com cells [a gift from J. F. Marshall (Barts Cancer Institute, Queen Mary University of London, London, England, UK)] were cultured in DMEM/F12 (Invitrogen, 11330-032) supplemented with 5% horse serum, epidermal growth factor (EGF; 20 ng/ml; PeproTech, AF-100-15), hydrocortisone (0.5 mg/ml; Sigma-Aldrich, H0888), cholera toxin (100 ng/ml; Sigma-Aldrich, C8052), and insulin (10 μg/ml; Sigma-Aldrich, I9278). HUVECs (Lonza, C2519A) were cultured on plasticware precoated with gelatin (0.1%; Sigma-Aldrich, G2500) for 10 min at 37°C. Subconfluent cells that were actively dividing were grown in endothelial cell growth basal medium 2 (Lonza, CC-3156) with SingleQuots (Lonza, CC-4176). HPMECs (PromoCell, C-12281) were cultured in endothelial cell growth mediumMV (PromoCell, C-22020). Experiments with HUVECs and HPMECs were conducted with monolayers where the medium was replaced with a reduced factor growth medium at least 24 hours before the experiment began or conditioned medium was collected: m199 medium (Sigma-Aldrich, M4530) supplemented with 20% FBS, l-glutamine (2 mM), endothelial cell growth factor supplements (30 mg/liter; Sigma-Aldrich, E2759), and heparin (10 U/ml; stock is 25 KU/ml in PBS; Sigma-Aldrich, H3393). HMEC-1 cells (ATCC, CRL-3243) were cultured in MCDB131 medium (Life Technologies, 10372019) with 100 mM l-glutamine, EGF (10 ng/ml; PeproTech, AF-100-15), and 10% FBS.

### Conditioned medium isolation and concentration

For conditioned medium experiments, medium was collected from cells after at least 24 hours and then concentrated using 3-kDa cut-off filters (Millipore, Amicon Ultra-15, UFC900324), centrifuging three times to first concentrate the medium and then washed twice with PBS. Protein concentration was measured with a Pierce detergent compatible Bradford assay kit (Thermo Fisher Scientific, 23246).

### Enzyme-linked immunosorbent assay for IGFBP2 or IGF-II in cell media

Enzyme-linked immunosorbent assays (ELISAs) were performed in a 96-well plate format using the human IGFBP2 SimpleStep ELISA kit (Abcam, ab215082) or human IGF-II/IGF2 Quantikine ELISA kit (DG200, R&D Systems), as per the manufacturer’s instructions.

### Flow cytometric analysis of IGFBP2 cell surface binding

MM231 cells were trypsinized, run through a single-cell filter, and then incubated with concentrated TIF medium (10 mg/ml) from either IGFBP2 or IGFBP2-Clover overexpressing TIFs for 30 min at 37°C. Next, cells were fixed with 2% PFA for 10 min at 37°C and washed two times with PBS. Between 50,000 and 100,000 cells were resuspended in 200 μl of PBS and loaded into a 96-well plate. Cytometry was then performed on an LSRFortessa cell analyzer using the High Throughput Sampler (BD Biosciences). The 488-nm laser power was adjusted using a positive control population of MM231 stably expressing IGFBP2-Clover before running the other conditions. Up to 10,000 single cells were collected per condition. Gating and statistical analysis of the cell population geometric means and robust SD were performed in FlowJo (BD Biosciences).

### Gene silencing

Gene silencing was performed by transfection of 10 nM siRNA using RNAiMAX, as per the manufacturer’s instructions. The nontarget control (NTC; AllStars negative control), *IGFBP2* siRNAs (Hs_IGFBP2_1, SI00012495, CACACGTATTTATATTTGGAA and Hs_IGFBP2_1, SI00012502, ACAGTGCAAGATGTCTCTGAA), and *IGF2* siRNAs (Hs_IGF2_8, SI04949441, CAGGGTAAACTGGCCATCCGA and Hs_INS_IGF2_4, SI03495485, CCGGTCCTCTTTATCCACTGT) were all purchased from QIAGEN.

### Generation of stable cell lines

Lentiviral particles were produced in human embryonic kidney 293FT packaging cells (Thermo Fisher Scientific, R70007) by cotransfecting with the third-generation lentiviral packaging system, pMDLg/pRRE (Addgene, plasmid #12251), pRSV-Rev (Addgene, plasmid #12253), and pMD2.G (Addgene, plasmid #12259), along with the pLenti6.3/TO/V5-DEST-mTurquoise2, pLenti6.3/TO/V5-DEST-IGFBP2, and pLenti6.3/TO/V5-DEST-IGFBP2-Clover transfer plasmids using Lipofectamine 3000 (Thermo Fisher Scientific) in OptiMEM (Gibco, 31985070), as per the manufacturer’s protocol. Twenty-four hours after transfection, the medium was replaced with TIF growth medium and incubated for a further 24 hours, at which point the medium was collected and filtered through a 0.45-μm syringe filter. MM231s or TIFs were then transduced with the lentiviral particles for 48 hours, in the presence of polybrene (8 μg/ml; Sigma-Aldrich, TR-1003-G), before washing and selecting stable positive cells using puromycin (1 μg/ml).

### Immunofluorescence

Staining of cultured cells was performed after fixation with 4% PFA (Thermo Fisher Scientific, 28908)/0.3% Triton X-100 for 10 min at 37°C. Samples were then washed with PBS before blocking overnight with 2.5% BSA (Sigma-Aldrich, A8022)/1 M glycine (ITW Reagents, A1067)/PBS. Blocked samples were then incubated overnight at 4°C with primary antibodies against human IGFBP2 (Santa Cruz Biotechnology, sc-25285), PPARγ (1:100; PA3-821A, Thermo Fisher Scientific, rabbit), paxillin (Y113; 1:100; ab32084, Abcam), or vimentin (D21H3; 1:100; rabbit, 5741, Cell Signaling Technology). Primary antibodies were then removed and the samples were washed with PBS before staining with secondary antibodies and/or Nile Red (10 μM, 19123-10MG, Sigma-Aldrich; stock: 1 mM in methanol), SiR-actin (Tebu-Bio, SC001), and/or 4′,6-diamidino-2-phenylindole (DAPI; D1306, Life Technologies).

For adhesion experiments in the presence of recombinant IGFBP2, 35-mm glass-bottom dishes were coated with 10 μg/ml of both fibronectin (Millipore, 341631; stock: 1 mg/ml) and collagen I (Millipore, 08-115; stock: 4 mg/ml) overnight at 4°C. MM231 cells were then seeded for 1.5 hours before fixation and staining, as above. Stained samples were then imaged on a spinning disk confocal microscope (3i Marianas CSU-W1; 20×/0.8 objective) and analyzed for staining intensity, focal adhesion, or cell area in FIJI (NIH).

### Immunohistochemistry

Organotypic matrices and xenografts were fixed in 10% neutral buffered formalin and then paraffin embedded, before cutting 4-μm sections for staining with either hematoxylin and eosin (H&E) or primary antibodies against Ki67 (1:500; Dako, M7240) or pan-cytokeratin (1:25; Invitrogen, MA5-13203). Slides were then scanned on a Pannoramic P1000 (3DHISTECH, 20×/0.8 objective).

Immunofluorescence of formalin-fixed paraffin-embedded (FFPE) sections was performed with standard protocols on deparaffinized sections after heat-mediated antigen retrieval in universal buffer (Retriever 2100, Aptum Bio) with the indicated antibodies against vimentin (1:500; D21H3; Cell Signaling Technology, 5741) or IGFBP2 (1:100; Santa Cruz Biotechnology, sc-25285). All samples were stained with DAPI (Life Technologies), mounted in Mowiol containing 1,4-diazabicyclo[2.2.2]octane (DABCO; Merck) antifade reagent.

For immunofluorescence of frozen tissue sections, samples were fixed overnight at +4°C in periodate-lysine-PFA buffer [1% PFA, 0.01 M sodium periodate, 0.075 M l-lysine, and 0.0375 M P-buffer (0.081 M Na_2_HPO_4_ and 0.019 M NaH_2_PO_4_) (pH 7.4)]. After washing twice with P-buffer, samples were incubated in 30% sucrose (Merck, 107687) in P-buffer for a minimum of 2 days. Samples were mounted in Tissue-Tek O.C.T. Compound (Sakura, 4583) on dry ice and cut into 8-μm sections. The frozen sections were thawed for 1 hour at room temperature (RT) before immunolabeling. The sections were blocked and permeabilized in 2% BSA and 0.1% Triton X-100 in PBS for 30 min at RT. Primary antibodies—vimentin (1:500; D21H3; Cell Signaling Technology, 5741), IGFBP2 (1:100; Santa Cruz Biotechnology, sc-25285), keratin-8 (1:1000; Hybridoma Bank, clone Troma-I), or keratin-14 (1:1000; Covance, PRB-1558)—were incubated in 2% BSA in PBS overnight at +4°C. The sections were washed 3 × 10 min with PBS. The fluorescently conjugated secondary antibodies were incubated in 2% BSA in PBS for 1 hour at RT, after which the sections were washed 3 × 10 min with PBS (1:1000; DAPI in the second wash) and then 5 min with milli-Q water. The sections were mounted under a glass coverslip with Mowiol (Calbiochem) supplemented with 2.5% DABCO (Sigma-Aldrich). Labeled sections were imaged on a spinning disk confocal microscope (3i Marianas CSU-W1; 20×/0.8 objective). Autofluorescence was from 488-nm excitation and detecting 525-/50-nm emission. For the quantification of IGFBP2 intensity in FIJI (NIH), adipocytes were identified on the basis of morphological characteristics using the autofluorescence channel.

### Invasion into fibroblast-contracted 3D collagen I matrices

Fibroblast-contracted “organotypic” 3D collagen I matrices for invasion assays were generated as described previously (*9*), modifying the protocol only to place MM231-seeded matrices in Transwell inserts (8-μm pore size; Greiner, ThinCert, 662638), after being cut to fit with a scalpel. These inserts were placed in 24-well wells containing preformed HUVEC or adipocyte monolayers (adipocytes were differentiated in these wells before Transwell addition) on the bottom of the well. An air-liquid interface was then formed by adding media up to the bottom of the Transwell insert. In experiments without HUVEC/adipocyte monolayers, only the culture medium was added up to the bottom of the Transwell insert. After 14 days of invasion, matrices were carefully removed from the Transwells and fixed in 10% neutral buffered formalin overnight at 4°C, then paraffin embedded, sectioned, and stained as described above. Quantification of the proliferation or invasion indices was performed in QuPath using the positive cell detection algorithm over eight regions of interest. For invasion assessment, the fraction of cells in a region of interest that had invaded beyond 100 μm was compared to the total number of cells in that region.

### Inverted invasion assay

Inverted invasion assays were performed as described previously (*62*). After 5 days, MM231 cells were fixed in 4% PFA/0.2% Triton X-100 (T8787, Sigma-Aldrich) in PBS for 1 hour at RT, washed three times in PBS, before incubation with phalloidin-488 (Invitrogen, A12379) overnight at 4°C, and then imaged on a Zeiss LSM880 inverted confocal microscope using a 40×/1.2 objective and taking *z*-stacks with 15-μm increments. Invasion indices were calculated in FIJI (NIH) by measuring the area of signal in increments beyond 45 μm and then dividing this by the total area of the signal in all of the 15-μm increments to get a percentage of invading cells beyond 45 μm. For the exogenous protein inverse invasion screen, cells were treated with either PBS or one of the following recombinant proteins (5 μM): SPARC (R&D Systems, 941-SP), DKK1 (R&D Systems, 5439-DK), DKK3 (R&D Systems, 1118-DK), TFPI (R&D Systems, 2974-PI), TFPI2 (R&D Systems, 2545-PI), HHIP (R&D Systems, 9280-HP), PAI1 (R&D Systems, 1786-PI), IGFBP2 (R&D Systems, 674-B2), IGFBP7 (R&D Systems, 1334-B7), and GSN (Novus, H00002934-Q01). For assessment of the IGF-II pathway perturbation, the following were used: recombinant IGF-II (10 ng/ml; R&D Systems, 292-G2), anti–IGF-II (10 μg/ml; Sigma-Aldrich, 05-166), or immunoglobulin G1κ (IgG1κ; 10 μg/ml; Sigma-Aldrich, M7894).

### Mass spectrometry

MM231 cells were treated with concentrated conditioned media from TIFs overexpressing mT2 or IGFBP2-Clover (~30 μg). For the mT2 control medium, ~30-μg recombinant GFP (Abcam, ab84191) was added. After 1 hour, medium was collected and fixed with Dimethyl dithiobispropionimidate (DTBP) (5 mM; Thermo Fisher Scientific, 20665). Fixation occurred at 37°C for 30 min with gentle mixing. This was then quenched by the addition of quenching buffer [500 mM tris-HCl (pH 8.0) and 750 mM NaCl] for a further 10 min at 4°C while gently mixing. Once quenched, GFP-trap beads (30 μl, Chromotek, gfa) were added to the medium and incubated overnight at 4°C while gently mixing. Immunoprecipitations were then spun down at 300*g* for 5 min before washing beads thrice with PBS and then once with 50 mM tris-HCl (pH 8.0) and 150 mM NaCl. For the on-bead digestion, samples were reduced with 10 mM dithiothreitol (DTT)/50 mM tris-HCl (pH 8.0) in the presence of 8 M urea for 1 hour at 37°C and then alkylated in 40 mM iodoacetamide/50 mM tris-HCl (pH 8.0) for 1 hour in the dark. DTT was then added in excess to consume the remaining alkylating agent and dilute the urea before overnight digestion with trypsin (Promega, V5111) at 37°C. Samples were then acidified with trifluoroacetic acid (pH ~2) before desalting using Sep-Pak C18 96-well plates (Waters, 186002321). Liquid chromatography–tandem mass spectrometry was then performed as described previously (*63*), modified only in the use of 300 ng of sample for the analysis and in the application of a 50-min two-step gradient from 5 to 21% of eluent B for 28 min to 36% of eluent B for 22 min, followed by a wash stage with 100% of eluent B to eluate peptides. Assignment of peptides and quantification of abundance ratios (normalized to total peptide amount) by label-free quantification was performed in Proteome Discoverer 2.5 (Thermo Fisher Scientific) using intensity values from the precursor ions.

### Matrigel invasion assay

Matrigel invasion chambers (354480, Corning) or Matrigel-coated Transwells (60 μl per Transwell; allowed to set for 1 hour at 37°C; Greiner, ThinCert; 8-μm pore size, 662638) were seeded with 2 × 10^5^ MM231, DCIS.com, or MM468 cells per chamber in 500-μl serum-free medium. The bottom chamber was then filled with 1-ml complete medium and either PBS, recombinant IGFBP2 (5 μM; R&D Systems, 674-B2), anti–IGF-II (10 μg/ml; Sigma-Aldrich, 05-166), or IgG1κ (10 μg/ml; Sigma-Aldrich, M7894). After allowing invasion to occur for 16 hours, the invasions were then fixed with 4% PFA in PBS. The cells in the top chamber were removed with a cotton tip and then the cells were stained with DAPI before imaging on a spinning disk confocal microscope (3i Marianas CSU-W1; 10×/0.45 objective).

### Molecular cloning

To generate the lentiviral constructs pLenti6.3/TO/V5-DEST-IGFBP2 (Addgene, #191006), pLenti6.3/TO/V5-DEST-IGFBP2-Clover (Addgene, #191008), and pLenti6.3/TO/V5-DEST-mTurquoise2 (Addgene, #191010), entry clones were LR subcloned with the Gateway destination vector pLenti6.3/TO/V5-DEST (Invitrogen) using LR Clonase II (Invitrogen, 11791), as per the manufacturers’ protocol. pENTR221-IGFBP2-Clover (Addgene, #191007) was synthesized by BioCat (Heidelberg, Germany), while pENTR2b-mTurquoise2 (Addgene, #191014) was generated by first polymerase chain reaction (PCR)–amplifying mT2 from pmTurquoise2-N1 [Addgene, #54843 (*64*)] using primers 5′-GGCTGGCGCCGGTACCGCCACCATGGTGAGCAAGGGCG-3′ and 5′-GGGTCTAGATATCTCGAGTCATTACTTGTACAGCTCGTCCATGCCGAGAG-3′. This PCR fragment was then digested with Xho I/Kpn I (New England Biolabs), in parallel with digestion of the pENTR2b (Invitrogen, A10463) backbone with the same restriction enzymes. These fragments were then ligated using T4 DNA ligase (Thermo Fisher Scientific, EL0011). All plasmids were validated by analytical digests and sequencing.

### Proliferation assays

To assess the proliferation in cancer cells after siRNA gene knockdown or through treatment with recombinant proteins or conditioned media, parallel 96-well plates were seeded, and a single plate was assessed on each day of the assay using cell counting kit-8 (Sigma-Aldrich, 96992), as per the manufacturer’s instructions. Relative cell density was measured as absorbance at 450 nm after a 4-hour incubation with the cell counting kit-8 reagent at 37°C. Day 0 was measured immediately after the cells had seeded down in the wells, before any treatment. Doubling times were obtained through fitting an exponential growth equation to the data using Prism 7 (GraphPad Software).

### Patient samples

Human breast tissue samples were obtained at the Department of Plastic and General Surgery at Turku University Hospital (Turku, Finland) with approval from the Ethics Committee of the Hospital District of Southwestern Finland (permit number 23/1801/2018) and with a written consent from the patients (§279, 9/2001). Healthy breast samples were obtained from eight female patients undergoing reduction mammoplasty surgery (ages 18 to 45; data S2). Breast tumors of nine female patients with breast cancer (ages of 41 to 85) were excised and examined by a clinical pathologist (data S2). Tissue samples were processed to frozen tissue sections or FFPE tissue sections and H&E labeled with standard procedures.

### Primary human cell isolation and adipocyte culture

Adipocytes were differentiated from the stromal cells of healthy breast tissue obtained from reduction mammoplasty operations. The tissue was transferred in transport medium [DMEM/F12 GlutaMAX (Gibco, 10565018) supplemented with 5% FBS (Sigma-Aldrich, F7524) and 10 mM Hepes (Sigma-Aldrich)]. Extra fat was removed, and the tissue was dissected into pieces of ~1 mm^3^ for enzymatic digestion in dissociation medium [penicillin/streptomycin (1:100), 5% FBS, filter-sterilized collagenase (300 U/ml; type XI, Sigma-Aldrich, C7657), and hyaluronidase (100 U/ml; type I-S, Sigma-Aldrich, H3506) in mammary epithelial cell growth medium (PromoCell, C-21010)] at 37°C with rotation overnight. The dissociated glandular tissue was centrifuged for 5 min at 600*g*. The cell pellet was then resuspended in 10 ml of transport medium with deoxyribonuclease I (DNAse I; 10 μg/ml), shaken occasionally for 1 min to digest DNA, pelleted, and resuspended again in 10 ml of transport medium. The tissue was then pulse centrifuged two times for 1 min and two times for 30 s at 80*g*. After each pulse centrifugation, the supernatant containing the mammary stromal cell fraction was collected, pooled, and preserved at −150°C. For experiments, mammary stromal cells were thawed and plated on 6-well plates (Thermo Fisher Scientific), 24-well plates (Cellstar), or μ-Slide 8 well (Ibidi, 80826) in subcutaneous preadipocyte medium (PM-1; ZenBio) for 2 to 3 days to obtain preadipocytes. For differentiation initiation, PM-1 was carefully changed to subcutaneous preadipocyte differentiation medium (DM-2; ZenBio), and cells were kept in DM-2 for 1 week without medium change. Last, DM-2 was changed to subcutaneous adipocyte maintenance medium (AM-1; ZenBio). After a week of culture in AM-1, preadipocytes were fully differentiated to mature adipocytes with visible lipid droplets. For maintenance of preadipocyte control cells, cells were kept in PM-1 that was changed every other day.

### Recombinant protein purification

BL21 competent *Escherichia coli* (New England Biolabs, C2530H) were transformed with pET28a-CNA35-mCherry [Addgene, plasmid #61607 (*65*)] plasmid DNA and grown overnight on a shaker at 37°C to yield a 250-ml culture with an OD_600_ (optical density at 600 nm) = 0.6. This was then incubated overnight again on a shaker at 30°C in the presence of isopropyl-β-d-thiogalactopyranoside (500 μM; Thermo Fisher Scientific, R0392). The bacteria were then pelleted by centrifugation at 6000*g* for 15 min at 4°C before discarding the supernatant and resuspending in 9-ml tris-buffered saline (TBS) with protease inhibitors (cOmpleteTM Mini, EDTA-free, Roche). To this solution, 1 ml of BugBuster (Millipore, 10× protein extraction reagent, #70921-50ML), 1 μl of benzonase nuclease (Sigma-Aldrich, E1014-5KU), 4.5 μl of DNAse I (Sigma-Aldrich, 11284932001), and lysozyme from chicken egg white (Sigma-Aldrich, 62970) were added. This mixture was then rotated for 30 min at 4°C before centrifugation at 6000*g* for 1 hour. The supernatant was then purified using a kit for His-tagged proteins (Macherey-Nagel, Protino Ni-TED2000 packed columns, #745120.25). The elution buffer was then exchanged against 4× changes of PBS using centrifugal filters (Millipore, Amicon Ultra-4, 10K UFC801024).

### RNA isolation, cDNA generation, and quantitative reverse transcription PCR

RNA from cultured cells was collected and isolated using the NucleoSpin RNA kit (Macherey-Nagel). For cDNA synthesis, 1 μg of the extracted RNA was then used as a template for the high-capacity cDNA reverse transcription kit (Applied Biosystems). Each PCR reaction was performed using 100 ng of cDNA and the appropriate TaqMan gene expression assays (with 6-carboxyfluorescein (FAM) dye label; Thermo Fisher Scientific) for each gene, according to the manufacturer’s instructions (Thermo Fisher Scientific, TaqMan Fast Advanced Master Mix, 4444557). The following TaqMan gene expression assays were used: IGFBP2 (Hs01040719_m1), complement factor D (Hs00157263_m1), adiponectin receptor 1 (Hs00360422_m1), PPARγ (Hs01115513_m1), lipase E (Hs00943405_g1), fatty acid binding protein 4 (Hs01086177_m1), and glyceraldehyde-3-phosphate dehydrogenase (GAPDH; Hs02786624_g1). Relative mRNA expression levels were normalized to GAPDH, and quantification was performed using the ΔΔCt method (*66*).

### Statistical analysis

Bar and line graphs are presented as means ± SEM of at least three independent experiments, where statistical significance is given by *P < 0.05, **P < 0.01, ***P < 0.001, or ns (not significant). All boxplots include min/max whiskers. The specific statistical tests applied are given in the respective figure legends. All statistical tests were performed in Prism 7 (GraphPad Software Inc.).

### Western blotting

Cell lysates were prepared in TXLB lysis buffer [50 mM Hepes, 1% Triton X-100, 0.5% sodium deoxycholate, 0.1% SDS, 0.5 mM EDTA, 50 mM NaF, 10 mM Na_3_VO_4_, and protease inhibitor cocktail (cOmplete Mini, EDTA-free, Roche)], and volumes were adjusted according to protein concentration measurements (DC protein assay kit, Bio-Rad, 5000111). Separation was performed by gel electrophoresis (Mini-PROTEAN TGX Precast Gels 4-20%, Bio-Rad, 4561096), before transferring onto a nitrocellulose membrane (Trans-Blot Turbo Transfer System, Bio-Rad) and blocking with AdvanBlock-Fluor (Advansta, R-03729-E10). Primary antibodies in AdvanBlock-Fluor were incubated overnight at 4°C; IGFBP2 (R&D Systems, AF674), IGF-II (Millipore, 05-166-MI), GAPDH (Hytest, 5G4MAB6C5), and GFP (Thermo Fisher Scientific, A11122). Membranes were washed between primary and secondary antibody treatments with Tris-buffered saline with 0.1% Tween 20 (TBST). IRDye secondary antibodies (1:5000 diluted in TBST; LI-COR) were incubated for at least 1 hour at RT, before detection on an Odyssey fluorescence imager CLx (LI-COR). Densitometry analysis was performed in FIJI (NIH) by normalizing the signal to GAPDH, which was used as a loading control.

## Notes

View/request a protocol for this paper from *Bio-protocol*.
