# Supplementary material for: IGFBP2 secretion by mammary adipocytes limits breast cancer invasion
Source: Sci Adv. 2023 Jul 12;9(28):eadg1840. doi: 10.1126/sciadv.adg1840 (PMC10337915; doi:10.1126/sciadv.adg1840)
Supplement: Supplementary file 1 — Figs. S1 to S5 Legends for data S1 to S3 [file sciadv.adg1840_sm.pdf]

Science Advances
AAAS

Supplementary Materials for

IGFBP2 secretion by mammary adipocytes limits breast cancer invasion

James R. W. Conway *et al.*

Corresponding author: James R. W. Conway, jdconw@utu.fi; Johanna Ivaska, joivaska@utu.fi

Sci. Adv. **9**, eadg1840 (2023)
DOI: 10.1126/sciadv.adg1840

The PDF file includes:

Figs. S1 to S5
Legends for data S1 to S3

Other Supplementary Material for this manuscript includes the following:

Data S1 to S3

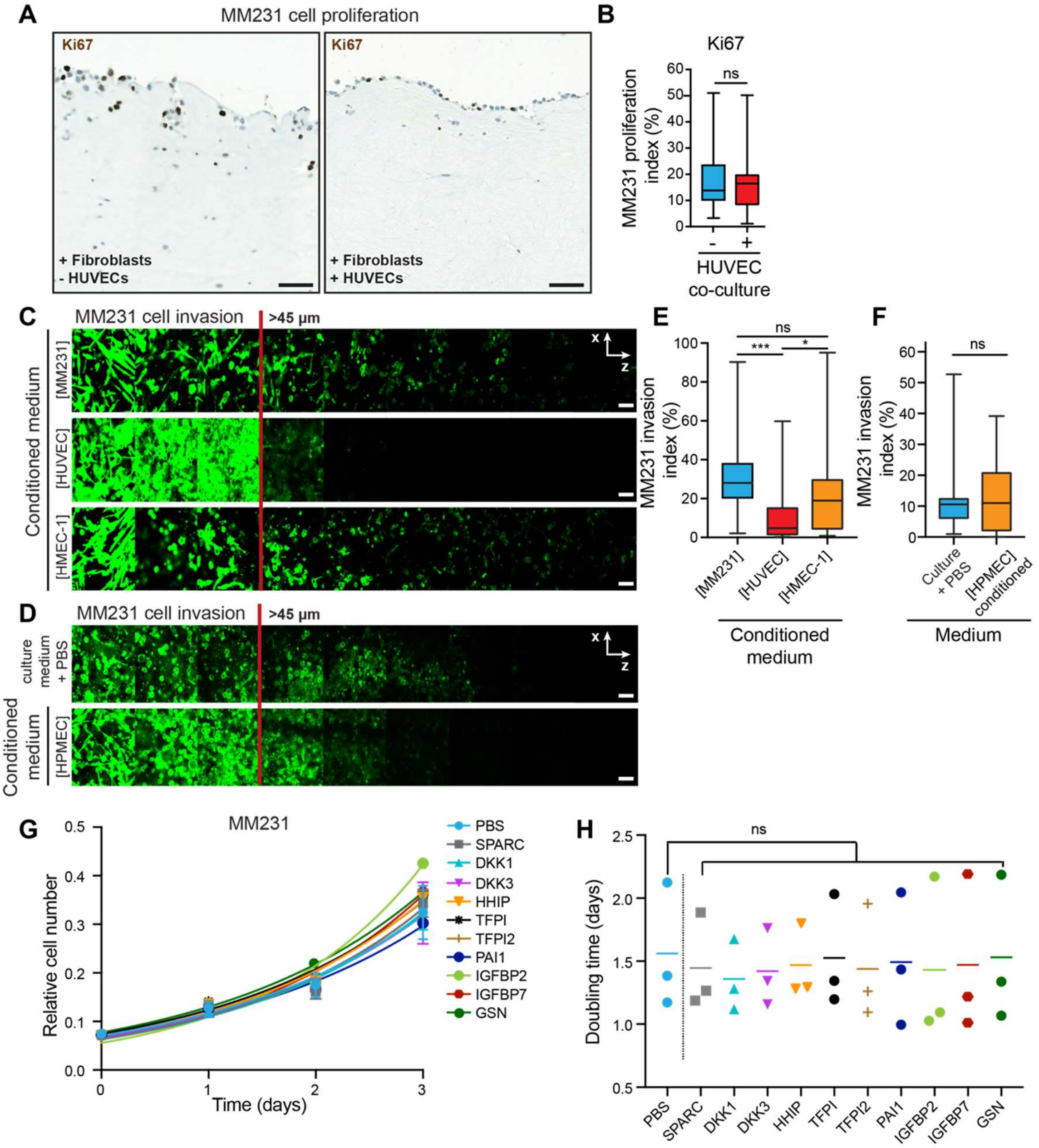

Fig. S1. HUVEC co-culture and secreted factors had no significant effect on MM231 proliferation.

(**A** and **B**) Representative images (A) and quantification (B) of MM231 cell proliferation (stained with Ki67) during invasion into fibroblast-contracted 3D collagen matrices ± HUVEC co-culture, performed in the presence of endothelial growth factor-reduced medium. Scale bars, 50 µm. (n = 3 biological replicates, triplicate matrices, 8 regions/condition/replicate; one-way ANOVA with a Tukey correction; *p<0.05, **p<0.01, ***p<0.001, ns – not significant). (**C** to **F**) Representative images (C and D) and quantification (E and F) of MM231 breast cancer cell invasion in inverted collagen/fibronectin matrices in the presence of concentrated conditioned media (given in square brackets) from either MM231, HMEC-1 or HUVEC cells (C) or from HPMEC cells, compared to medium with the same volume of PBS as a control (D). Scale bars, 50 µm. (n=3 biological replicates performed in triplicate; one-way ANOVA

with a Tukey correction (E); two-tailed Student's t-test with a Welch's correction (F); *p<0.05, ***p<0.001, ns – not significant). (**G** and **H**) Representative curves (G) and doubling times (H) from the relative cell density of MM231 cells treated with the given recombinant secreted factors (5 μM) on day 0, 1, 2, 3, against PBS as a control (Performed with 5 wells/treatment condition; n=3 biological replicates; one-way ANOVA with a Dunnett correction; ns – not significant).

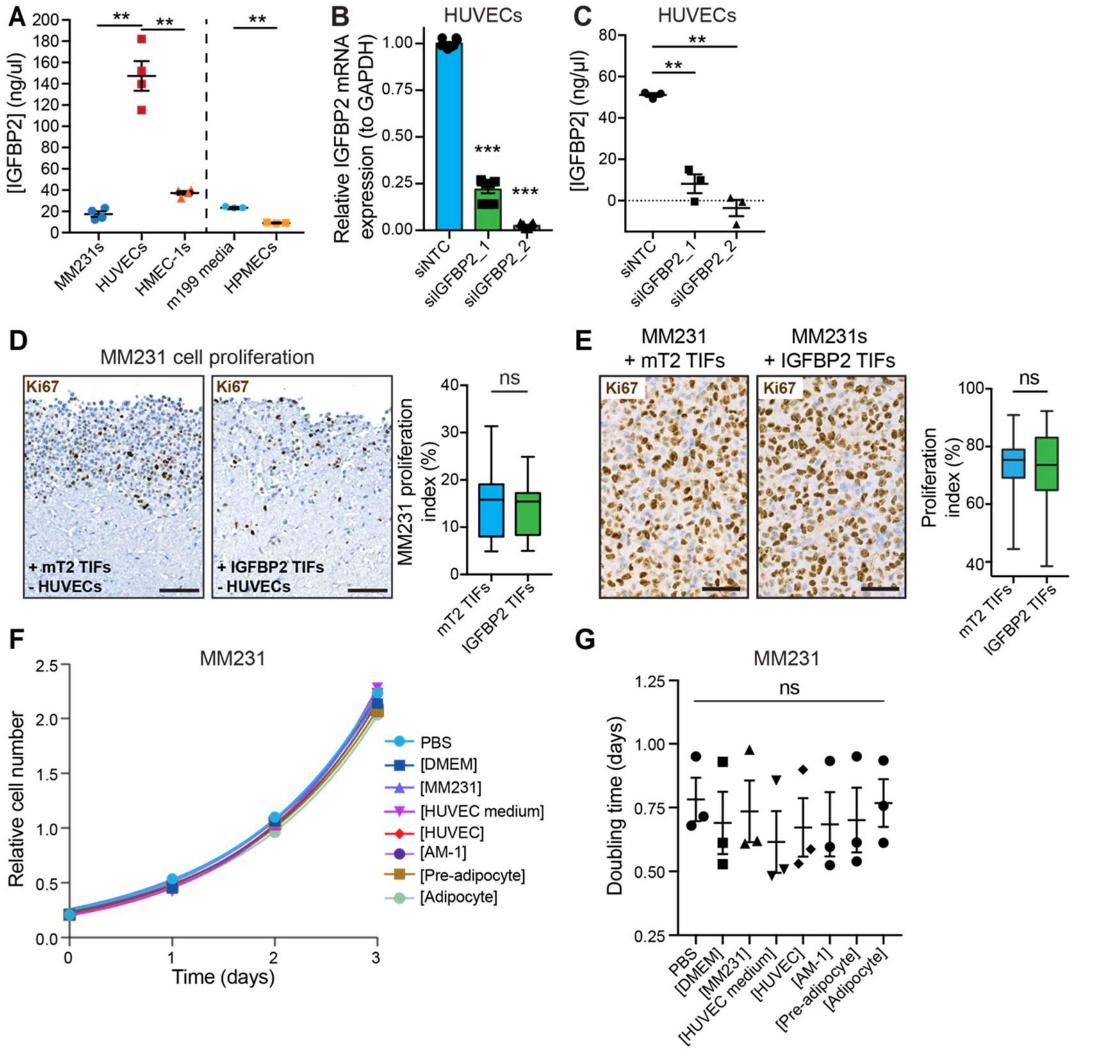

Fig. S2. Neither conditioned media, nor TIF-derived IGFPB2, had any significant effect on MM231 proliferation.

(**A**) ELISA assays for human IGFBP2 from conditioned media from MM231s, HUVECs, HMEC-1s, and from m199 media and HPMECs (n = 3-4; one-way ANOVA with a Tukey correction; **p<0.01). (**B**) Assessment of relative mRNA levels by qRT-PCR of RNA isolated from HUVECs after transfection with siRNAs against *IGFBP2* (siIGFBP2_1 and siIGFBP2_2) and siNTC (n=3; one-sample t-test; ***p<0.001). (**C**) ELISA assays for human IGFBP2 from conditioned media measured in duplicate (n=3; one-way ANOVA with a Dunnett correction; ***p<0.001). (**D**) Representative images and quantification of Ki67 stained organotypic invasions from mT2 or IGFBP2 TIF-contracted matrices, where MM231 cancer cells invaded for 14 days and are positive for Ki67 (brown nuclei if actively proliferating; n=3 biological replicates, triplicate matrices, 8 regions/condition/replicate; one-way ANOVA with a Tukey correction; ns – not significant). Scale bars, 100 µm. (**E**) Representative images of Ki67 stained MM231 xenografts, co-injected with TIFs overexpressing either mT2 (control) or IGFBP2. Quantification of positive (brown) to negative (blue) staining of Ki67 in 400 µm^2 regions of interest from subcutaneous xenografts (n=10 (mT2) and 12 (IGFBP2); two-tailed Student's t test with a Welch's correction; ns – not significant). Scale bars, 50 µm. (**F and G**) Representative curves (F) and doubling times (G) from the relative cell density of MM231 cells treated with conditioned media (given in square brackets) from MM231, HUVEC, pre- and mature adipocytes, against their respective concentrated growth medias as controls (Performed

with 3 wells/treatment condition; n = 3 biological replicates; one-way ANOVA with a Dunnett correction; ns – not significant).

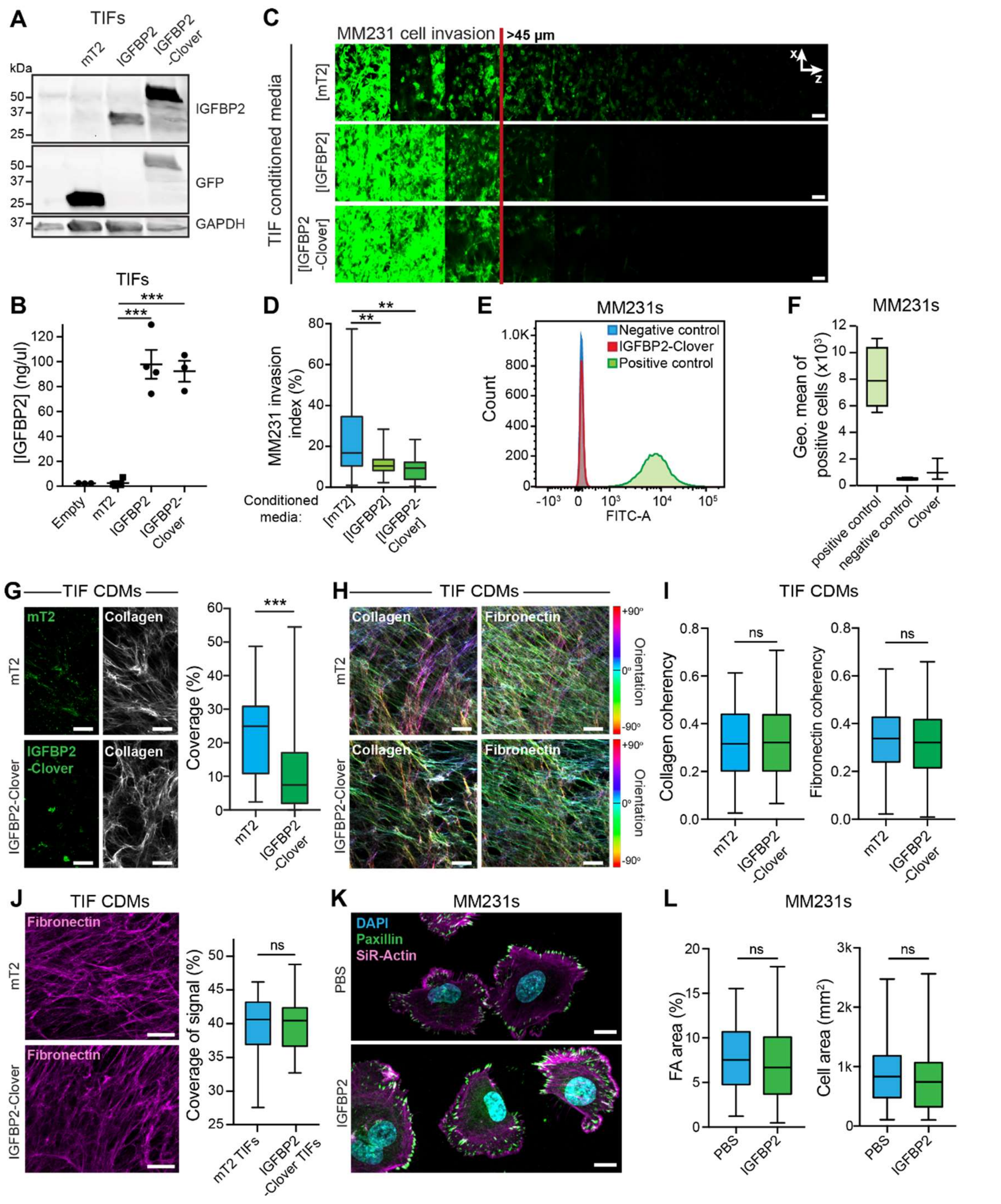

Fig. S3. IGFBP2 does not bind to the cancer cell surface or ECM.

(**A**) Representative western blot of TIFs stably overexpressing mT2, IGFBP2 or IGFBP2-Clover. (**B**) ELISA assays for human IGFBP2 from conditioned media (n=3; one-way ANOVA with a Tukey correction; ***p<0.01). (**C** and **D**) Representative images (C) and quantification (D) of MM231 breast cancer cells invading into inverted collagen/fibronectin matrices in the presence of concentrated conditioned media (given in square brackets) from mT2, IGFBP2 or IGFBP2-Clover overexpressing TIF cells (n=3 biological replicates performed in triplicate with 3 stacks/transwell; one-way ANOVA with a Tukey correction; **p<0.01). Scale bars, 50 μm. (**E** and **F**) Representative flow cytometry plot (E) and geometric means (F) from MM231 cancer cells expressing either IGFBP2-Clover (positive control) or treated with IGFBP2-

Clover exogenously (IGFBP2-Clover treatment), compared to treatment with exogenous untagged IGFBP2 (negative control; n = 3). (**G**) Representative images and quantification of signal coverage beyond an equal intensity threshold of CDMs derived from TIFs overexpressing either IGFBP2-Clover or mT2. The brightness of the mT2 and IGFBP2-Clover fluorescence channels was increased for visualization purposes to show the weak non-specific background signal in both (n=4, 8 ROI/condition/replicate; two-tailed Student's t-test with a Welch's correction; ***$p<0.001$). Scale bars, 50 μm. Images adjusted for display. (**H** and **I**) Representative images (H) and quantification of the coherency (I) from the fiber orientation analysis of the collagen I (CNA35-mCherry) or fibronectin labelling of CDMs from TIFs overexpressing either mT2 or IGFBP2. Scale bars, 50 μm. (n=4, 8 ROI/condition/replicate; two-tailed Student's t-test with a Welch's correction; *$p<0.05$, ns – not significant). (**J**) Representative images of fibronectin-stained CDMs derived from TIFs overexpressing either IGFBP2 or mT2, as well as quantification of the coverage of the fibronectin signal (n=4, 8 ROI/condition/replicate; two-tailed Student's t-test with a Welch's correction; *$p<0.05$, ns – not significant). Scale bars, 50 μm. (**K** and **L**) Representative immunofluorescence images (K) and quantification (L) of MM231 cell and focal adhesion (FA) area after seeding on a mixture of collagen/fibronectin in the presence or absence of IGFBP2 (5 μM); stained with DAPI, paxillin and SiR-Actin. Scale bars, 10 μm. (n = 3 biological replicates with >20 cells/replicate; two-tailed Student's t-test with a Welch's correction; ns – not significant).

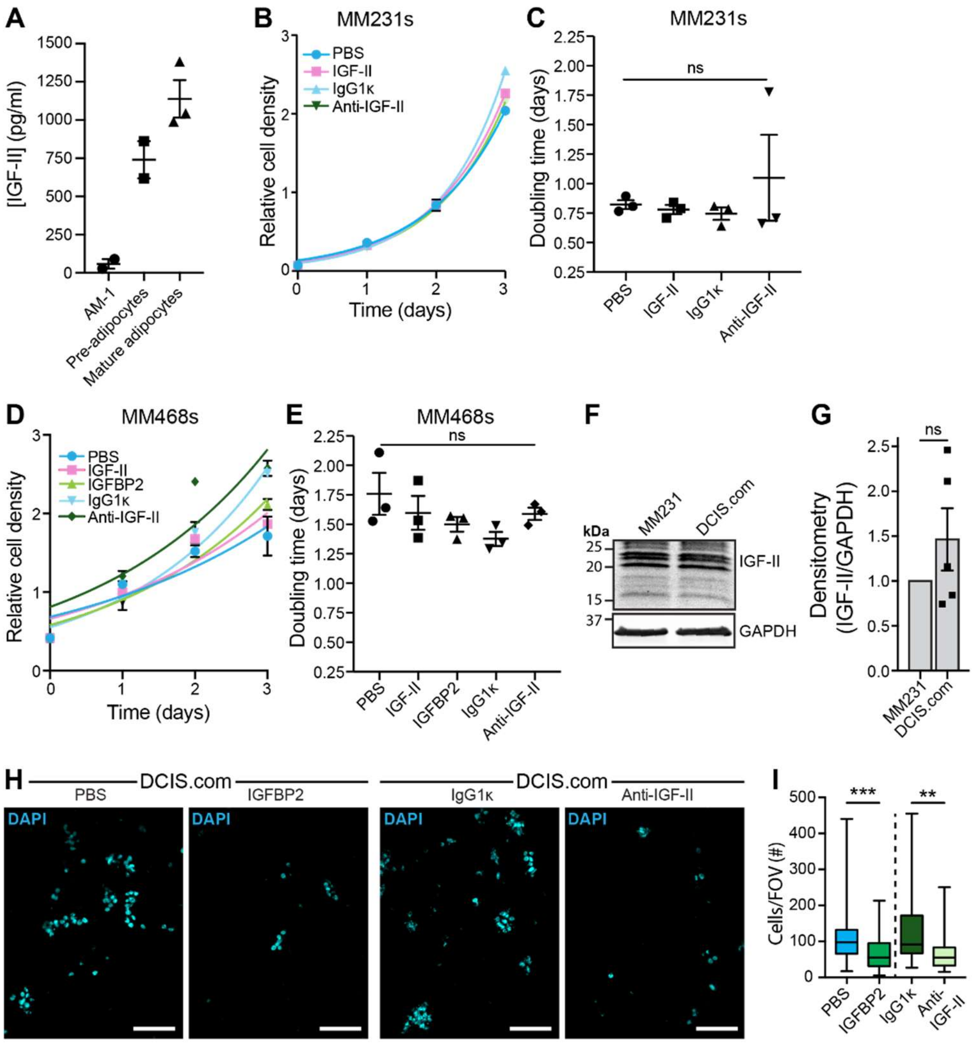

Fig. S4. IGF-II is important for DCIS.com invasion, while having no effect on MM231 and MM468 cell proliferation.

(**A**) ELISA assays for human IGF-II in conditioned media from pre- (n = 2 biological replicates) and mature adipocytes (n = 3 biological replicates), compared to the adipocyte culture media (AM-1). (**B** and **C**) Representative curves (B) and doubling times (C) from the relative cell density of MM231 cells treated with PBS, IGF-II (10 ng/ml), IgG (10 µg/ml) or anti-IGF-II (10 µg/ml; n = 3 biological replicates; one-way ANOVA with a Tukey correction; ns – not significant). (**D** and **E**) Representative curves (D) and doubling times (E) from the relative cell density of MM468 cells treated with PBS, IGF-II (10 ng/ml), IGFBP2 (5 µM), IgG (10 µg/ml) or anti-IGF-II (10 µg/ml; n = 3 biological replicates; one-way ANOVA with a Tukey correction; ns – not significant). (**F** and **G**) Representative IGF-II western blot (F) and quantification (G) from MM231 and DCIS.com cells (n = 5 biological replicates; one-sample t-test; ns – not significant). (**H** and **I**) Matrigel invasion assays for DCIS.com cells treated with PBS or IGFBP2, or IgG1κ or anti-IGF-II (n = 3, 8 fields of view (FOVs)/chamber, 2 invasion chambers/condition/replicate; two-tailed Student's t-test with a Welch's correction; ***p<0.001). Scale bars, 100 µm.

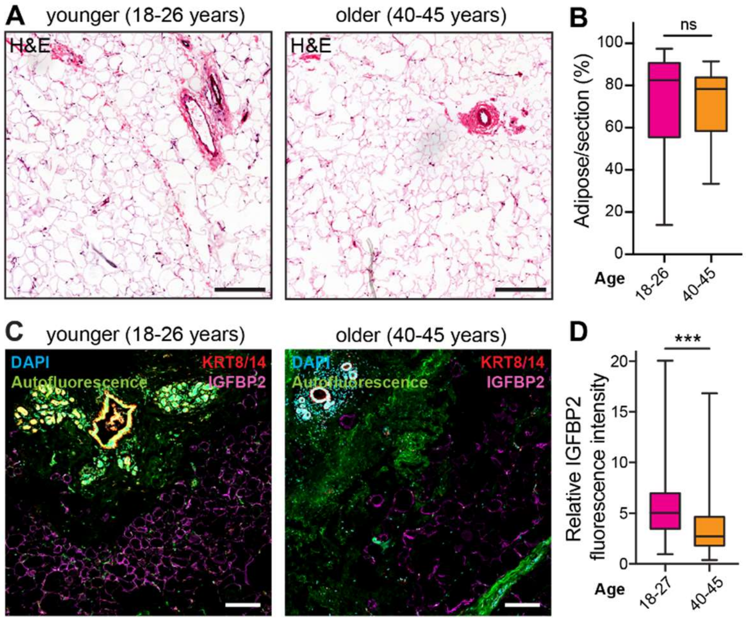

Fig. S5. IGFBP2 levels in mammary adipocytes are reduced in older patients.

(**A**) Representative H&E stained samples from younger (18-26 years; n = 4 patients) and older (40-45 years; n = 4 patients) healthy patient samples. Scale bars, 250 µm. (**B**) Quantification of adipocytes/section from older and younger patients (3-8 sections/patient; two-tailed Student's t-test with a Welch's correction; ns, not significant). (**C**) Representative images from patients stained for IGFBP2 (magenta) and counterstained with DAPI (cyan) and keratin-8/-14 (KRT8/14; red). Autofluorescence signal is given in green. Scale bars, 200 µm. (**D**) Quantification of IGFBP2 in adipocytes relative to unstained background (n = 4 normal reduction mammoplasty patient samples in each age group; 143-280 adipocytes/patient/group; two-tailed Mann-Whitney U test; ***$p<0.001$).

Data S1. (separate file "Data S1 - Secretomes.xlsx")
Comparison of four published HUVEC secretomes for common angiocrine factors.

Data S2. (separate file "Data S2 - Patient details.xlsx")
Patient details for those included in the study.

Data S3. (separate file "Data S3 - Mass spectrometry analysis.xlsx")
Analysis of GFP-trap mass spectrometry data for IGFBP2- or GFP-bound protein.
